# Supplementary material for: Genome-Wide Analysis, Modeling, and Identification of Amino Acid Binding Motifs Suggest the Involvement of GH3 Genes during Somatic Embryogenesis of Coffea canephora
Source: Plants (Basel). 2021 Sep 28;10(10):2034. doi: 10.3390/plants10102034 (PMC8539013; doi:10.3390/plants10102034)
Supplement: Supplementary file 1 [file plants-10-02034-s001.zip › Figures_Suppl.pdf]

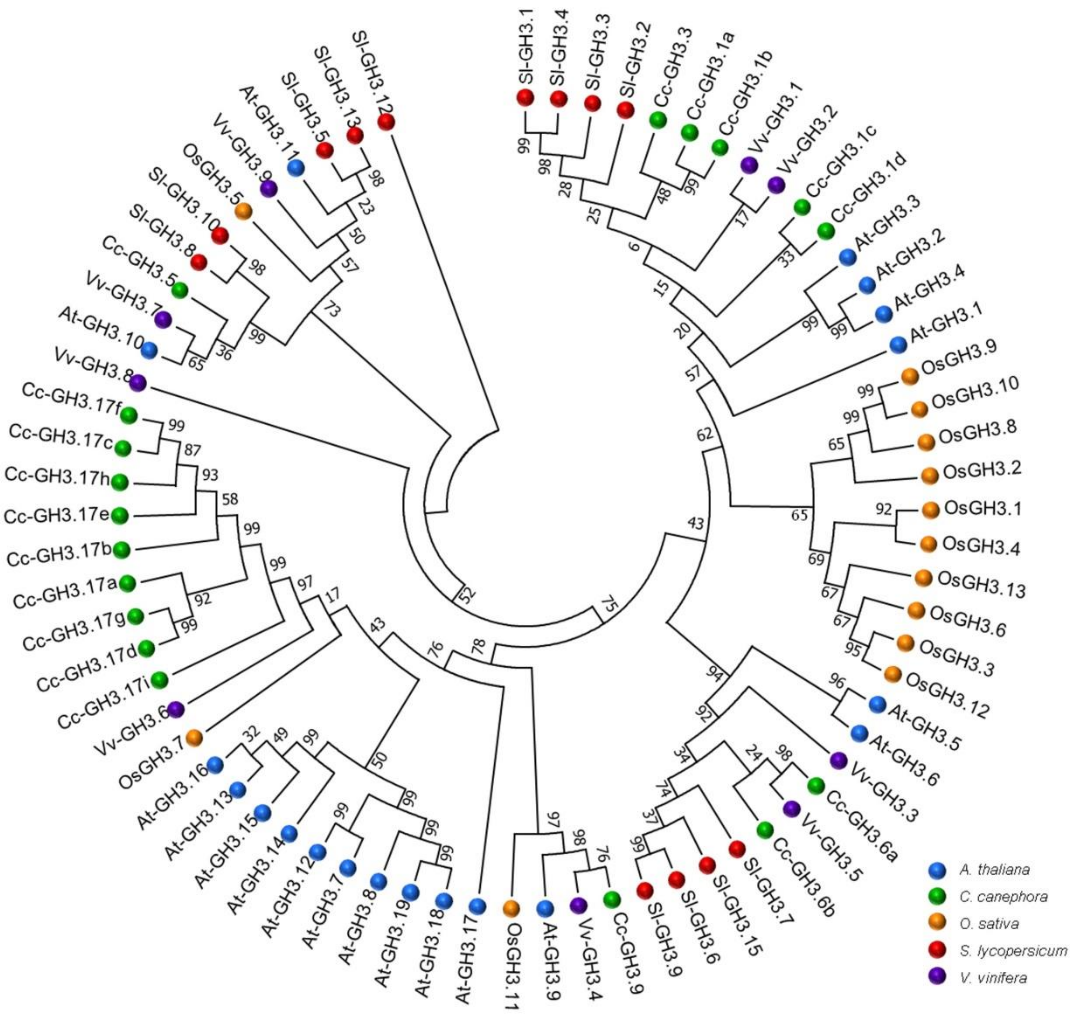


**Figure S1.** Analysis of the phylogenetic relationships of GH3 members in several species. A total of 19 sequences from *A. thaliana,* 18 from *C. canephora,* 15 from *S. lycopersicum,* 13 from *O. sativa*, and 8 from *V. vinifera* were used to construct a phylogenetic tree. GH3s belonging to one plant species are marked with the indicated leaf labels. The sequences were aligned in ClustalW, and analysis was conducted in MEGA7 using the Neighbor-Joining method. Bootstrap values (1000) are presented for all branches. All the GH3 members were clustered into different classes.

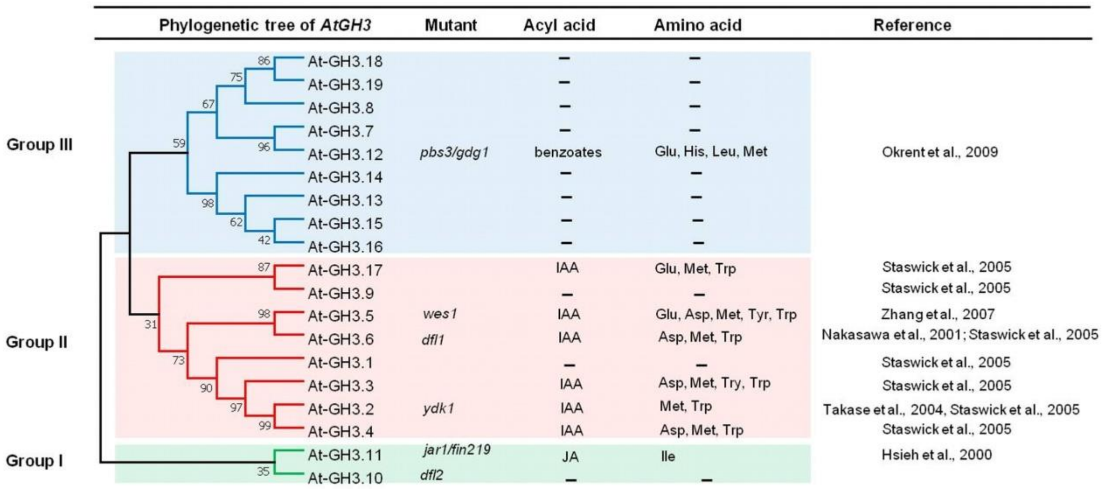


**Figure S2.** Phylogenetic relationship of the 19 GH3 genes reported in *A. thaliana*. Classification of groups I, II, and III, mutants associated with *AtGH3* and major acyl acid and amino acid substrates of the GH3 protein identified by substrate screening assays. The dashes in the table indicate unknown enzyme activity and substrate specificity.

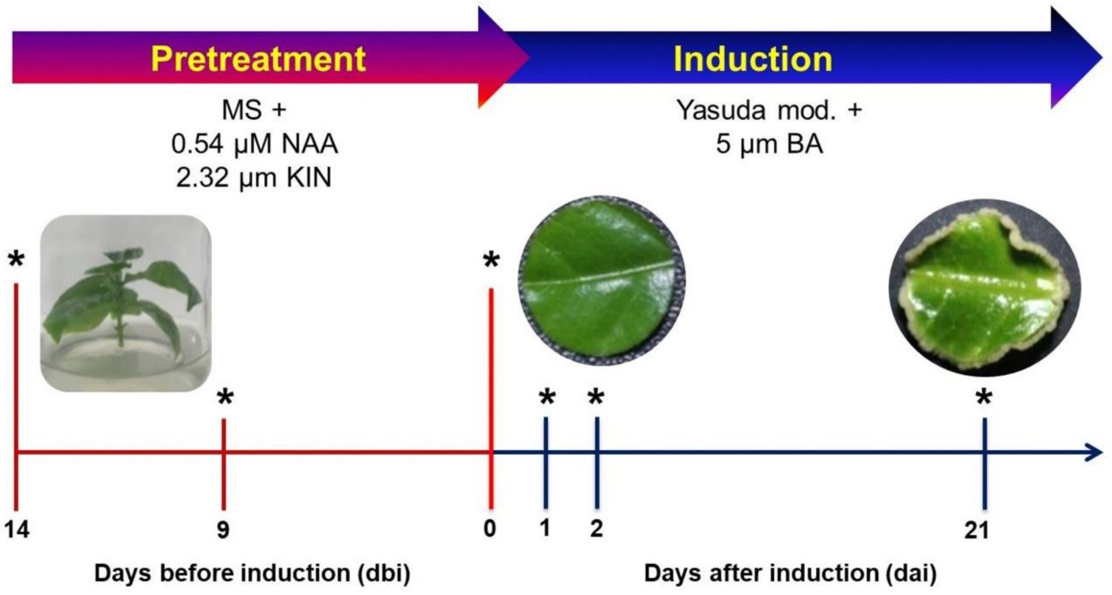


**Figure S3.** The SE induction process in *Coffea canephora* encompasses two stages: pretreatment and induction. First, the seedlings were preconditioned for 14 days in a semi-solid MS [63] medium supplemented with 0.54 µM NAA and 2.32 µM KIN under photoperiod conditions of 16 h light/8 h dark (150 µmol $m^{-2}s^{-1}$) at 25 ± 2 ºC. At the end of the pretreatment, the second and third pair of leaves were selected and cut into segments of 0.8 cm in diameter with the help of a sterile punch. Five explants were placed per 250 mL flask, with 50 mL of Yasuda liquid medium with the nitrogen source modified [42] and supplemented with 5 µM of BA, adjusted to pH 5.8 [37]. The flasks were incubated in the dark and shaking (55 rpm) at 25 ± 2 ºC for 56 days.
