# Supplementary material for: Genome-Wide Analysis, Modeling, and Identification of Amino Acid Binding Motifs Suggest the Involvement of GH3 Genes during Somatic Embryogenesis of Coffea canephora
Source: Plants (Basel). 2021 Sep 28;10(10):2034. doi: 10.3390/plants10102034 (PMC8539013; doi:10.3390/plants10102034)
Supplement: Supplementary file 1 [file plants-10-02034-s001.zip › Table S2.pdf]

Table S2. Gretchen Hagen (*GH3*) genes identified in some sequenced plant genomes.

Species	[1]Genome Size (Mb)	[2]Members of Group I	[3]Members of Group II	Members of Group III	Total members of GH3 family	Reference
Arabidopsis thaliana	120.69	2	8	9	**19**	[16]
Brassica napus	848.20	8	22	36	**66**	[23]
Brassica oleracea	554.98	4	9	12	**25**	[23]
Brassica rapa	234.69	4	10	24	**38**	[23]
Populus euphratica	496.033	4	6	4	**14**	[45]
Populus trichocarpa	434.29	4	6	3	**13**	[14]
Prunus persica	227.57	2	3	3	**8**	[14]
Triticum aestivum	14,471.4	10	10	16	**36**	[46]
Vitis vinifera	486.20	3	6	--	**9**	[21]
Zea mays	2243.62	5	8	--	**13**	[47]
Actinidia chinensis	653.93	4	11	--	**12**	[48]
Brachypodium distachyon	218.01	2	6	--	**8**	[49]
Carica papaya	370.42	2	4	--	**6**	[50]
Cicer arietinum	347.25	2	5	--	**11**	[51]
Coffea canephora	568.612	1	17	--	**18**	This study
Glycine max	1,116.18	8	16	--	**24**	[52]
Hordeum vulgare	233.13	2	7	--	**9**	[49]
Malus domestica	660.46	5	10	--	**15**	[53]
Medicago truncatula	429.61	6	7	--	**14**	[54]
Oriza sativa	490.15	4	9	--	**13**	[55]
Physcomitrella patens	472.08	2	--	--	**2**	[45]
Setaria italic	477.54	6	11	--	**17**	[49]
Solanum lycopersicum	812.994	9	6	--	**15**	[22]
Solanum tuberosum	772.25	4	9	--	**13**	[33]
Sorghum bicolor	374.25	4	7	--	**11**	[49]
Theobroma cacao	324.88	2	17	--	**19**	[52]
