# Supplementary material for: Genome-Wide Analysis, Modeling, and Identification of Amino Acid Binding Motifs Suggest the Involvement of GH3 Genes during Somatic Embryogenesis of Coffea canephora
Source: Plants (Basel). 2021 Sep 28;10(10):2034. doi: 10.3390/plants10102034 (PMC8539013; doi:10.3390/plants10102034)
Supplement: Supplementary file 1 [file plants-10-02034-s001.zip › Table S4.pdf]

**Table S4.** Gene-specific primer sequences used for Real-time qRT-PCR amplification.

| Gene | Forward primer | Reverse primer |
|---|---|---|
| *CcGH3.1a* | TGATCCAATATGCTCCCTTGCT | GTACAATCCGGCATGCGTAG |
| *CcGH3.1b* | GAGTTGTCAGTTGGGGTCCT | GCCCCTCTAGAGACAGCAAA |
| *CcGH3.1c* | CCGGGGTGCTAAACCCTAAA | CCCTGCCGACGCTGTAATAA |
| *CcGH3.1d* | AACCTTTACGTGCCGGGATT | GCAAAGCATTTGGGCGTACA |
| *CcGH3.3* | CTCGGACAAGACAGACGAGG | GTCCCGCTTTTTACCACACG |
| *CcGH3.5* | ATGCTGGGAACTTGCCTCTT | TTCTTGCCCCAGCTTGACTT |
| *CcGH3.6a* | CTGGCAGAAGTTTTAGAGGCTG | TTCTGAGGTCACAGGCAGGA |
| *CcGH3.6b* | GCTAGTGGCTCGTCCTGTTT | GGGTCGGTAATTCGTGGGTT |
| *CcGH3.9* | GCCGTATTTGCATCTGCGTT | CCCGTGATCACAGCCTCAAT |
| *CcGH3.17a* | GTCTCCTTGCTACTCTTTGAACTAA | CCGAGGCGAAAAATGTACCA |
| *CcGH3.17b* | CCTTGAACCACTTGGGCTCT | GCACGTTGAATGTTCTGATGC |
| *CcGH3.17c* | GCCTCGGGTTTGCTAAGGATA | TGGTCCTTGGCCAGATCTTC |
| *CcGH3.17d* | GAGCTACTGATCAGCTCGGG | CAAAGAACAGCGCTTTCCCA |
| *CcGH3.17e* | TGGTTCTTCAGAGGCGTTGT | GCATCTTTCCTGTTGGTGCAG |
| *CcGH3.17f* | GGATGGAGAGCCATCTCGGA | GCTTTCCCATCGTTCAAGCC |
| *CcGH3.17g* | CCACACTCCTGGTGGCTTAG | GCAAACCCGAGGCGAATATG |
| *CcGH3.17h* | GGAGCTTCAACTCAGATCAAACG | GTCCATGAGCACGTTGAATGT |
| *CcGH3.17i* | CAAAGTTGTGCAACGGCGTA | CCTCTCAAGCACTGCAGCTA |
| *Cyclophilin* | GATGGCGTGCCTCTTGGTAG | AGCCAAATTGCTTCCAGCCT |
