# Supplementary material for: Genome-Wide Analysis, Modeling, and Identification of Amino Acid Binding Motifs Suggest the Involvement of GH3 Genes during Somatic Embryogenesis of Coffea canephora
Source: Plants (Basel). 2021 Sep 28;10(10):2034. doi: 10.3390/plants10102034 (PMC8539013; doi:10.3390/plants10102034)
Supplement: Supplementary file 1 [file plants-10-02034-s001.zip › Table S5.pdf]

Table S5. Information of templates used for the construction of 3D proteins.

Protein	ID	Oligo-State	Sequence Identity	Coverage	QMEAN	Template	Method	Description
CcGH3.1b	Cc00_g01360	Monomer	60.69 %	0.98	-1.22	4b2g.1.A	X-ray, 2.40 Å	Crystal Structure of an Indole-3-Acetic Acid Amido Synthase from *Vitis vinifera* Involved in Auxin Homeostasis
CcGH3.5	Cc05_g06700	Monomer	36.09 %	0.96	-0.83	5kod.3.A	X-ray, 2.20 Å	Crystal Structure of GH3.5 Acyl Acid Amido Synthetase from *Arabidopsis thaliana*. Indole-3-acetic acid-amido synthetase GH3.5
CcGH3.6a	Cc05_g05640	Monomer	74.38 %	1.00	0.23	5kod.1.A	X-ray, 2.20 Å	Crystal Structure of GH3.5 Acyl Acid Amido Synthetase from *Arabidopsis thaliana*. Indole-3-acetic acid-amido synthetase GH3.5
CcGH3.6b	Cc05_g12940	Monomer	81.70 %	0.99	-0.06	5kod.2.A	X-ray, 2.20 Å	Crystal Structure of GH3.5 Acyl Acid Amido Synthetase from *Arabidopsis thaliana.* Indole-3-acetic acid-amido synthetase GH3.5
CcGH3.9	Cc01_g20620	Monomer	54.66 %	0.92	-0.66	7dk8.1.A	X-ray, 1.99 Å	Probable indole-3-acetic acid-amido synthetase GH3.8 Crystal structure of OsGH3-8 with AMP
CcGH3.17c	Cc00_g04520	Monomer	50.79 %	0.98	-1.45	6e1q.1.A	X-ray, 2.15 Å	AtGH3.15 acyl acid amido synthetase. AtGH3.15 acyl acid amido synthetase in complex with 2,4-DB
CcGH3.17d	Cc00_g04530	Monomer	50.35 %	0.98	-1.28	6e1q.1.A	X-ray, 2.15 Å	AtGH3.15 acyl acid amido synthetase. AtGH3.15 acyl acid amido synthetase in complex with 2,4-DB
